# Supplementary material for: Cryo-TEM structure of β-glucocerebrosidase in complex with its transporter LIMP-2
Source: Nat Commun. 2025 Mar 30;16:3074. doi: 10.1038/s41467-025-58340-1 (PMC11955523; doi:10.1038/s41467-025-58340-1)
Supplement: Supplementary file 1 — Supplelemtary Information [file 41467_2025_58340_MOESM1_ESM.pdf]

Cryo-TEM Structure of β-glucocerebrosidase in complex with its transporter LIMP-2

Supplementary Information

Supplementary Figures

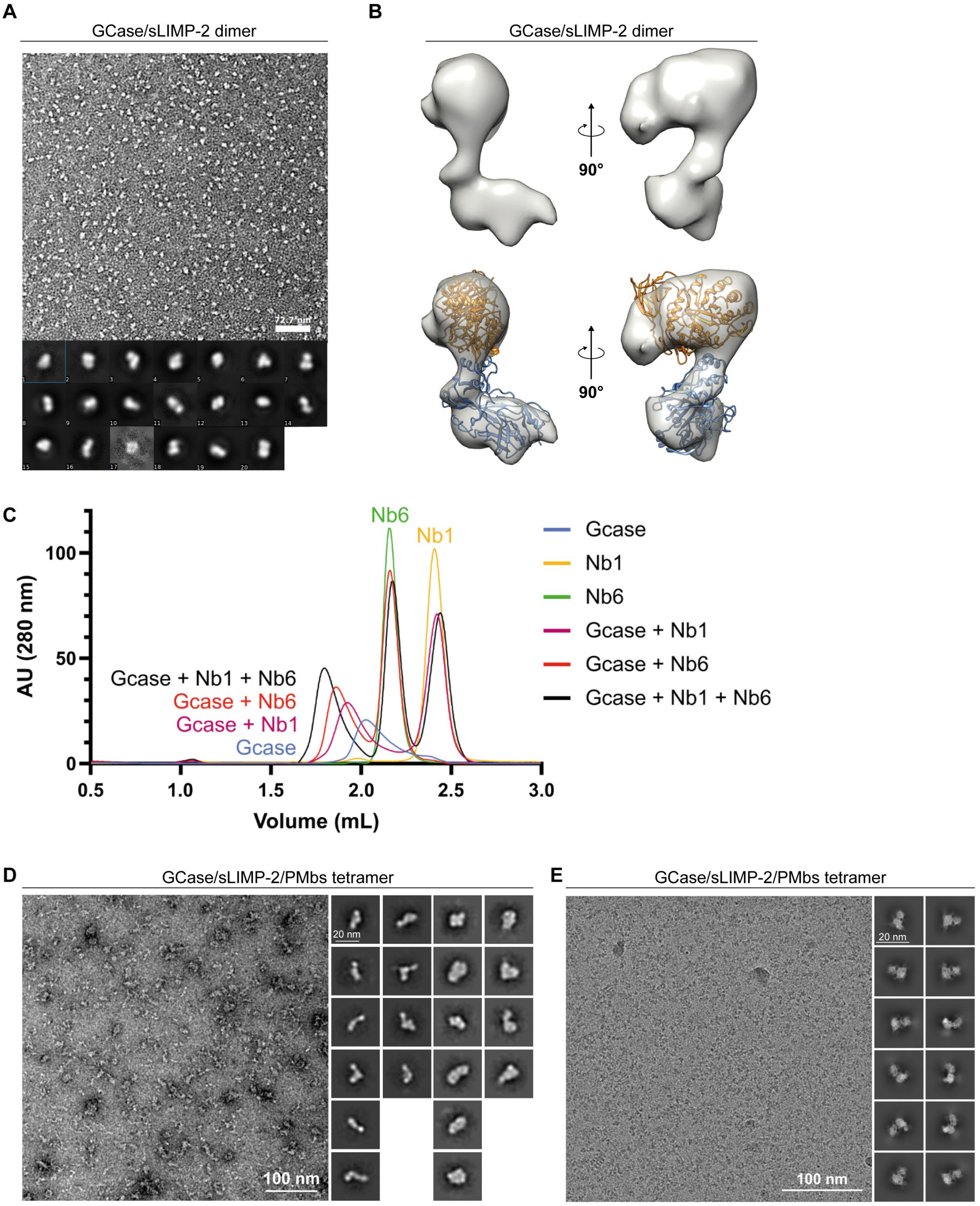

Supplementary Figure 1: Supporting data for sample preparation and EM analysis. A: Example micrograph and 2D classes from negative stain TEM of GCase/sLIMP-2 complex without PMbs. **B:** Low resolution map of GCase/sLIMP-2 complex reconstructed from negative stain data (A). For reference, the structures of GCase (orange) and LIMP-2 (blue) from the GCase/sLIMP-2/PMb complex were fitted into the low-resolution map to show the similar shape of the GCase/sLIMP-2 complex with and without PMbs. **C:** SEC chromatograms of recombinant GCase, Nb1 and Nb6. The chromatograms show one peak for GCase, representing the a GCase monomer. Addition of individual Nbs causes the GCase peak

to shift, confirming binding. Addition of both NBs yields a greater shift, confirming that both Nbs can bind simultaneously. Binding of Nbs did not produce any additional SEC peaks, suggesting no effect on GCase dimerization. **D, E:** Examples of micrographs and 2D classes of GCase/sLIMP-2/PMb complex from negative stain EM (D) and cryo-EM (E).

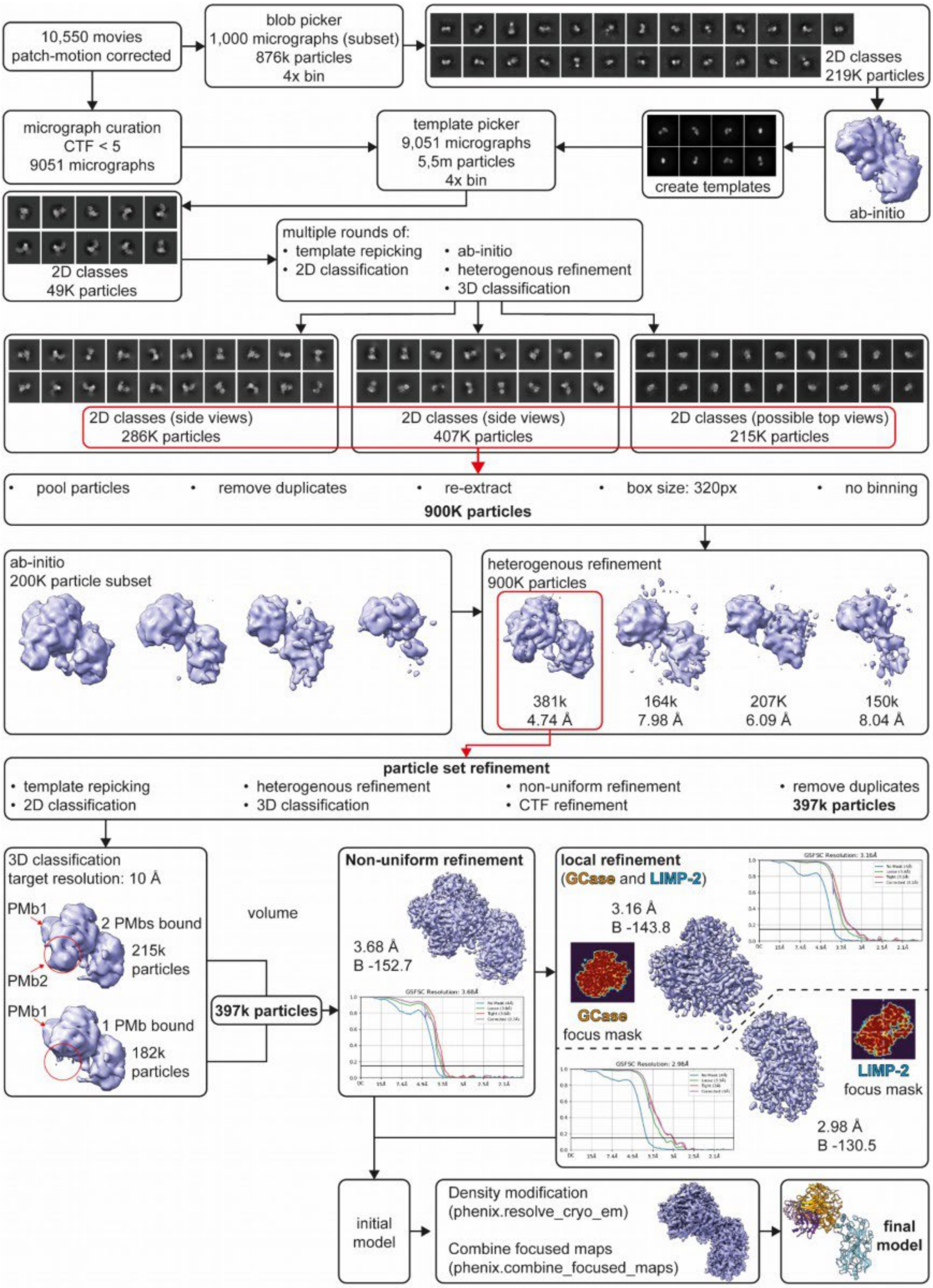

Supplementary Figure 2: Cryo-EM data processing workflow.

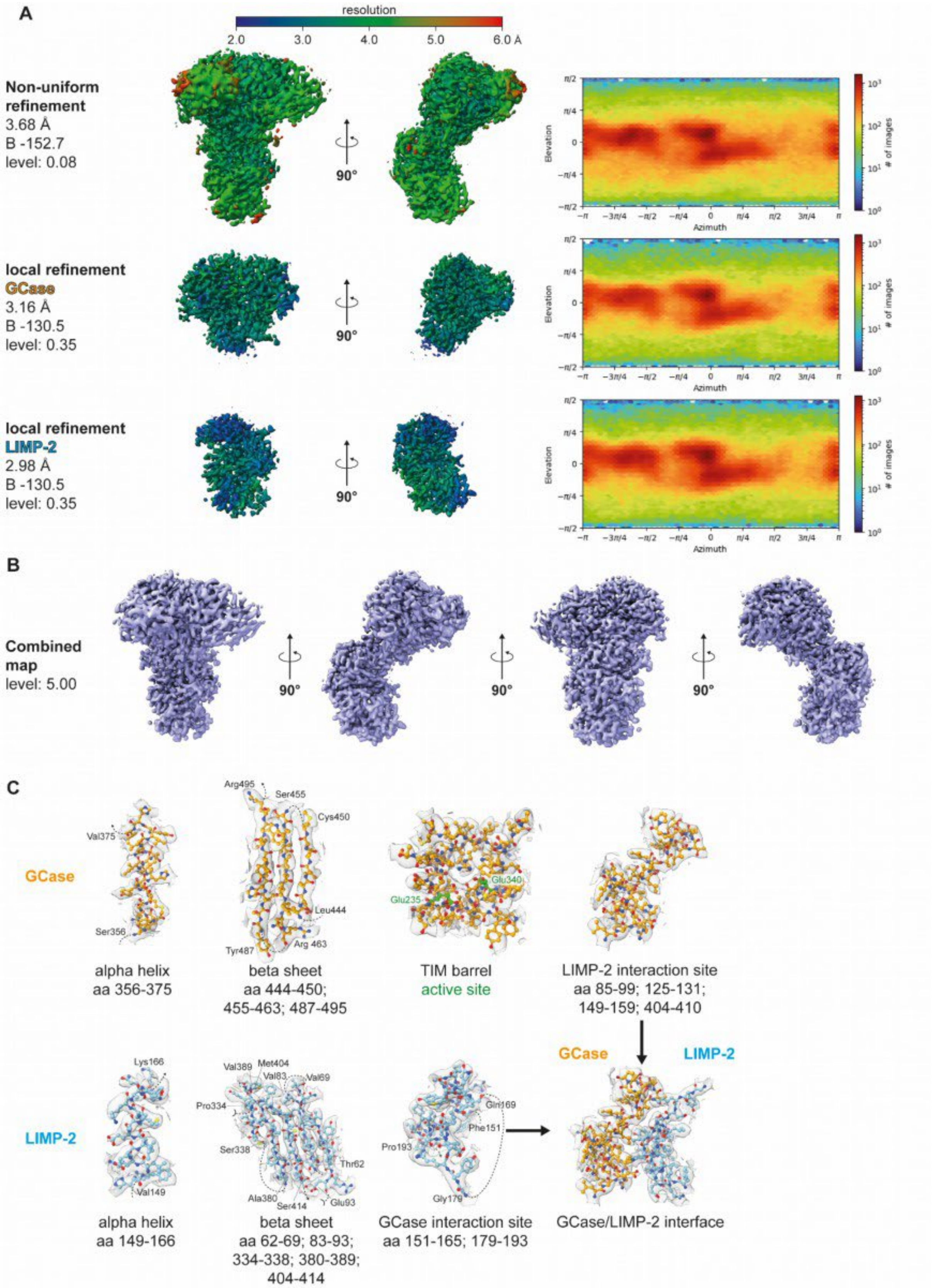

Supplementary Figure 3: Quality assessment of LIMP-2/GCase/PMb density map. A: Local resolution of obtained maps: non-uniform refinement (level: 0.08), GCase-focused local refinement (level: 0.35), LIMP-2-focused local refinement (level: 0.35). With angular distribution graphs. **B:** Overview of Combined map used for model refinement (level: 5.00). **C**: Local map quality of GCase (orange) and LIMP-2 (blue). Snippets show the density map (combined map, level: 5.00, surface zone: 3.00 Å around

shown residues) with the modeled protein for selected regions of GCase, LIMP-2 and the interaction interface.

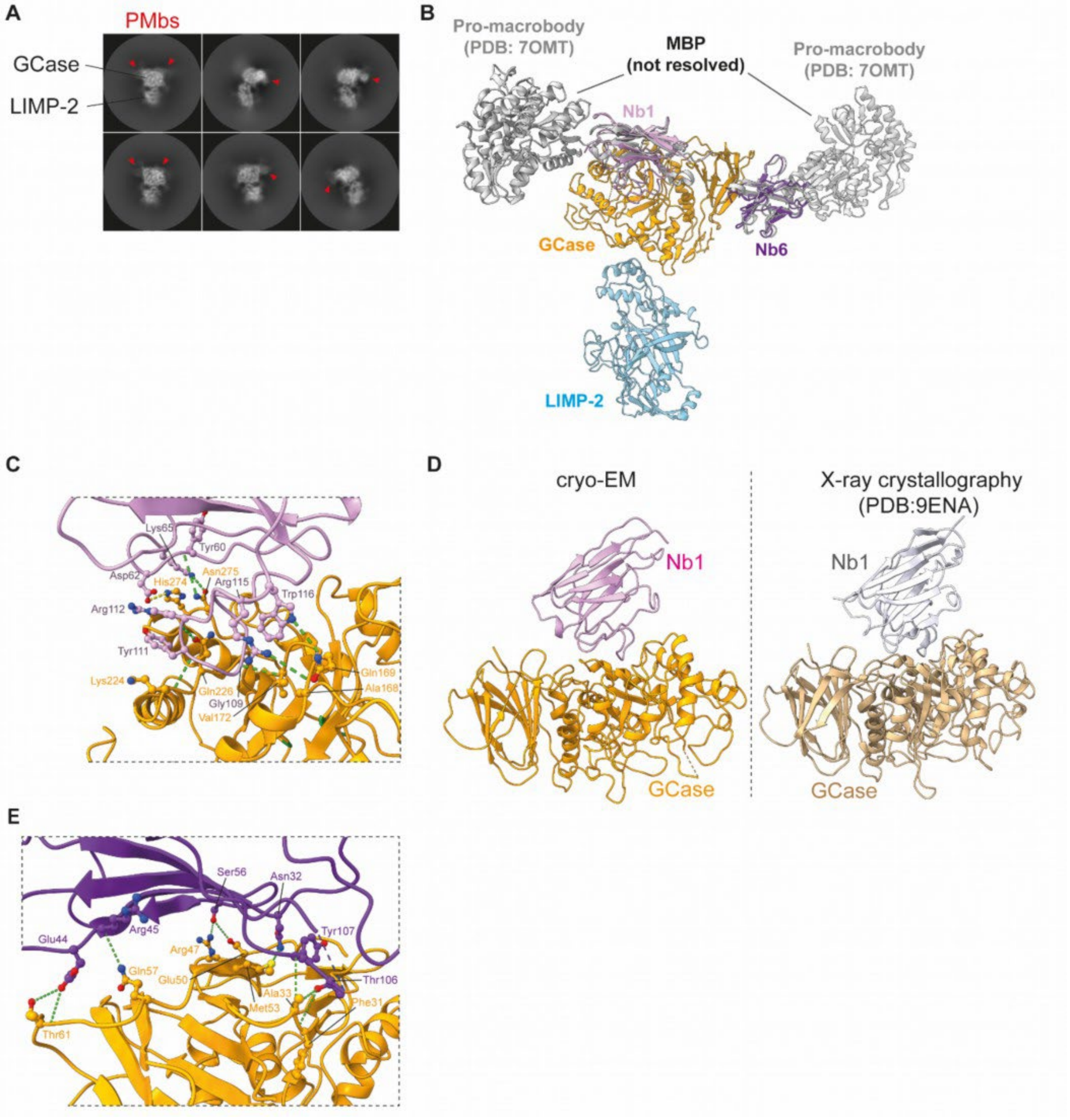

Supplementary Figure 4: Binding of PMbs to GCase. A: Selected 2D classes of LIMP-2/GCase/PMb tetramer showing bound Nbs (red arrows). No classes were observed where the MBP domains of the Nbs were resolved to more than diffuse density clouds. All classes were rotated to show GCase at the top and LIMP-2 at the bottom. **B:** Hypothesized orientation of the MBP domains (grey) of PMb1 and PMb2 when bound to the LIMP-2/GCase complex. The model was generated by structure superposition of Nb1 and Nb2 in the LIMP-2/GCase/Nb structure determined in this study with the high-resolution crystal structure of pro-macrobody 21 with bound maltose (PDB: 7OMT). **C:** Detailed view of the interaction interface of GCase and Nb1 showing residues interacting via hydrogen bonds (green) and salt bridges (yellow) as determined by PDBePISA. **D:** Side-by-side comparison of the cryo-EM structure of GCase-Nb1 obtained in this study and the previously published GCase-Nb1 crystal structure (PDB: 9ENA). **E:** Detailed view of the interaction interface of GCase and Nb2 showing residues interacting via hydrogen bonds (green) and salt bridges (yellow) as determined by PDBePISA.

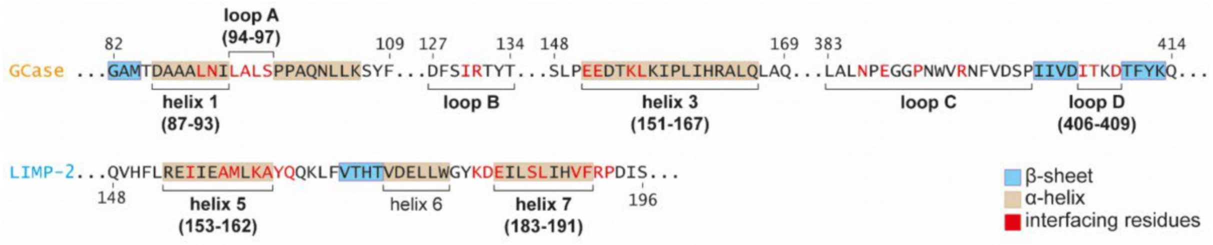

Supplementary Figure 5: Amino acid sequences of GCase (top) and LIMP-2 (bottom) participating in complex formation. Interaction domains of GCase (helix 1 and 3, loops A, B, C and D) and LIMP-2 (helix 5 and 7) are annotated and interacting amino acids as determined by PDBePISA are highlighted in red.

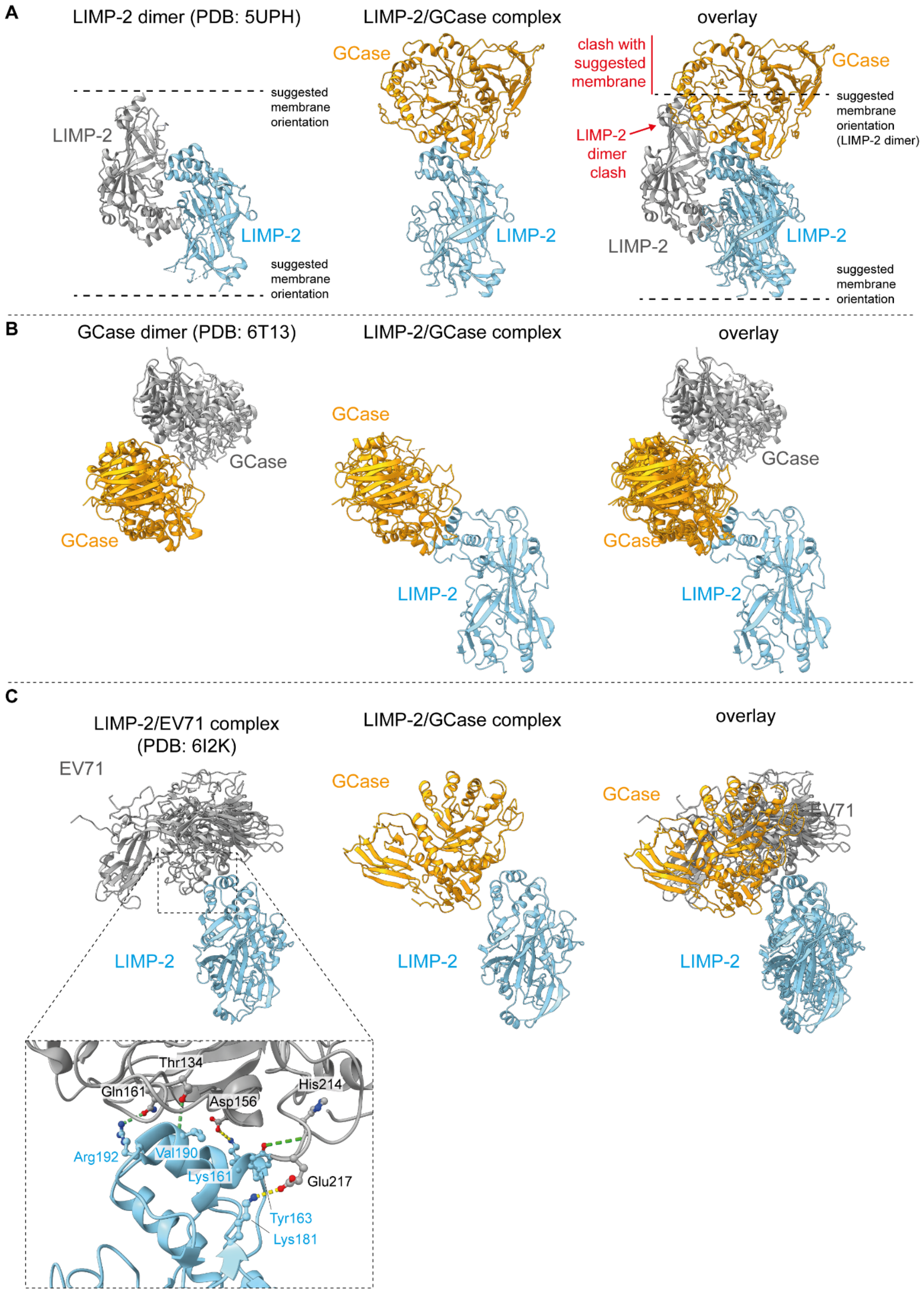

Supplementary Figure 6: Side-by-side structural comparisons of LIMP-2/GCase with previously published structures of known interaction partners. A: LIMP-2 homodimer (PDB: 5UPH); **B:** GCase homodimer (PDB:6T13); **C:** LIMP-2/EV71 complex (PDB: 6I2K) with detailed view of amino acids interacting via hydrogen bonds (green) and salt bridges (yellow) as determined by PDBePISA.

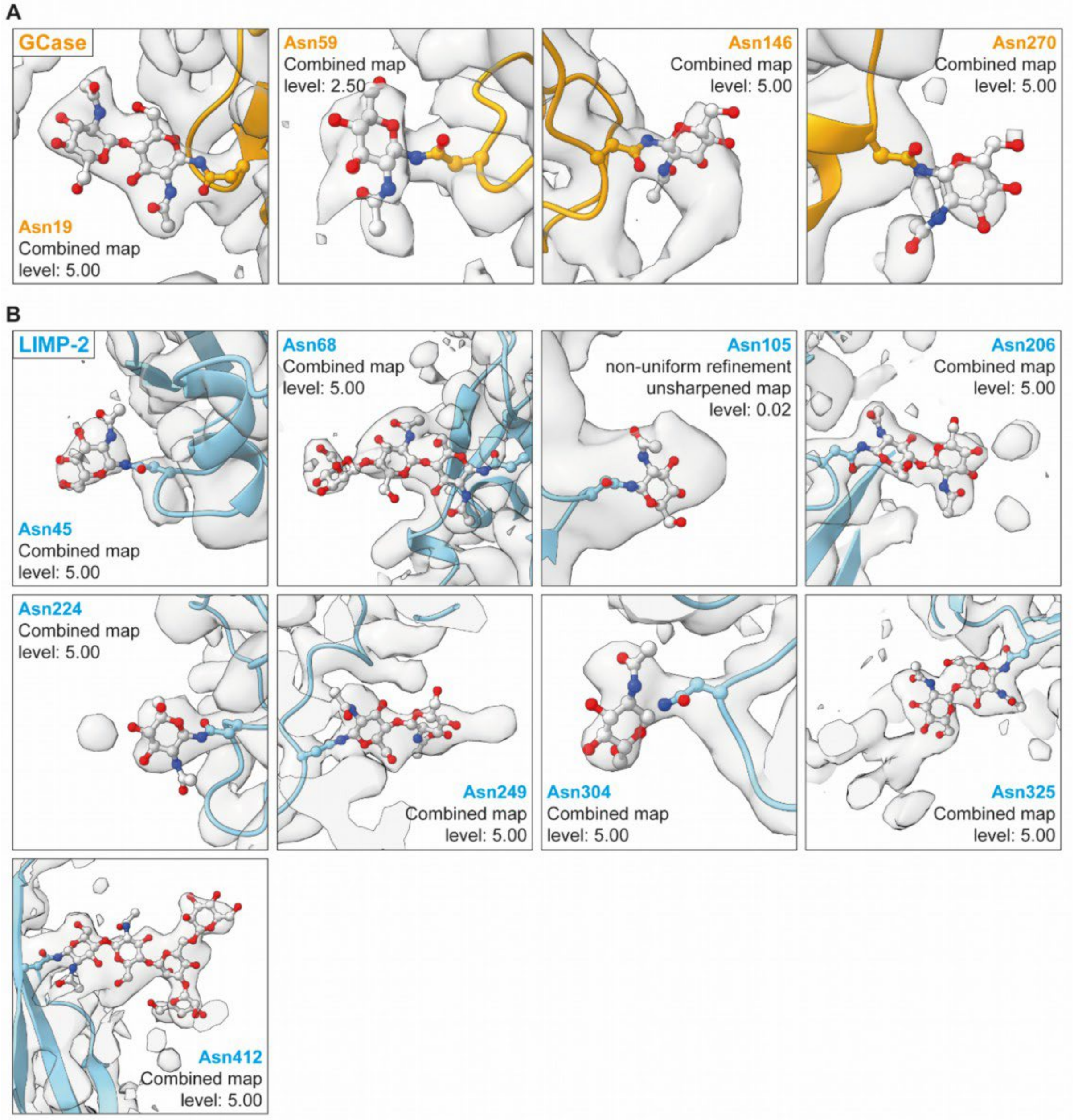

Supplementary Figure 7: N-glycosylation sites on GCase (A) and LIMP-2 (B). A screenshot is shown of each asparagine residue where additional density was observed in either the combined map or the unsharpened map obtained via non-uniform refinement (only for Asn105). Additional density can be attributed to the presence of N-linked glycans, which are not fully resolved due to high flexibility. The first sugar residues of each tree were modeled to the best degree that the obtained maps allowed.

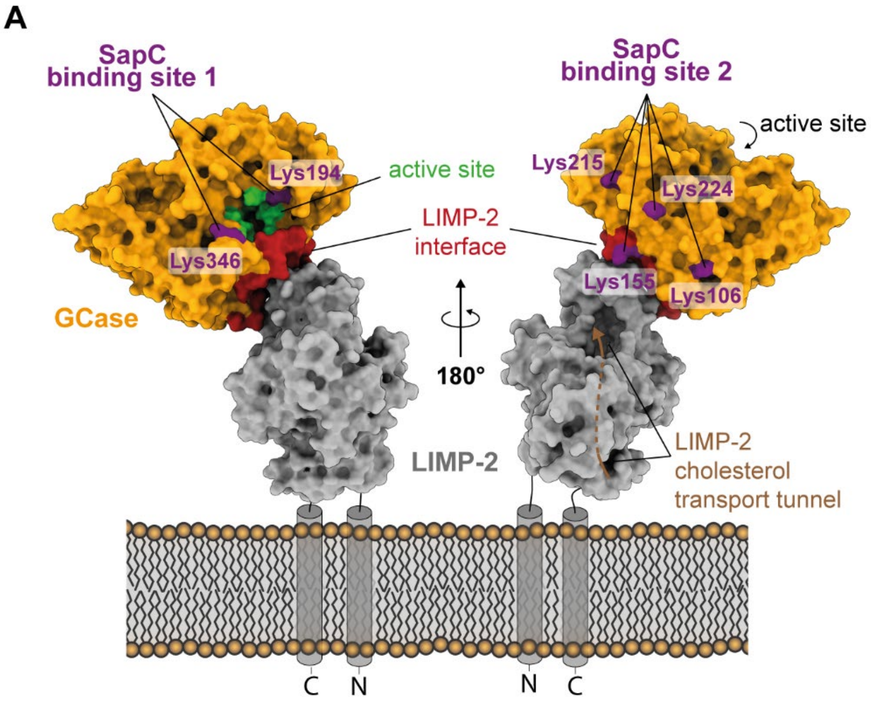

Supplementary Figure 8: Potential Saposin C (SapC) binding motives. Experimentally determined SapC interaction sites 1 and 2 with GCase. Binding sites base on cross-linking experiments and mass-spectrometry assisted peptide identification (lysine residues in purple). None of the reported binding sites would interfere with the GCase/LIMP-2 complex. A detailed list of GCase-SapC interacting residues can be found in Tab S4. Lysine 157 (Lys157) is hidden.

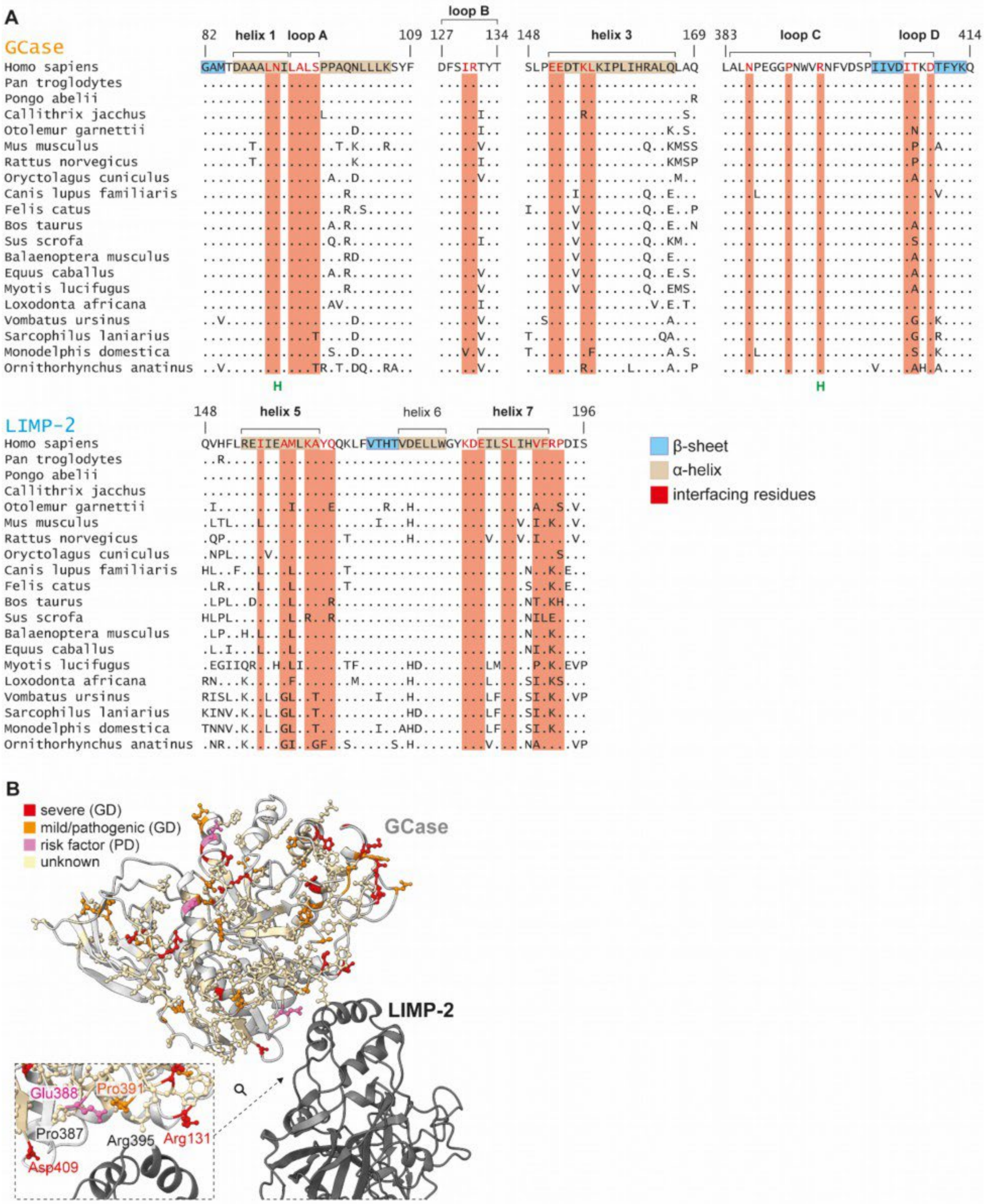

Supplementary Figure 9: Conserved amino acids in GCase/LIMP 2 interface. A: Conservation of human GCase/LIMP-2 interface regions in mammals. Conservation plots show the amino acid sequences of the human GCase/LIMP-2 interface regions as well as the conservation in respective homologs. Amino acids are shown in the one-letter code and periods indicate evolutionarily conserved residues in the respective homologs of human GCase and LIMP-2. Amino acid exchanges are represented by the one-letter code. Interacting amino acids as determined by PDBePISA (Figure S5) and the homologous positions are highlighted in red. Residue numbers of the human proteins are depicted on top of the human sequence regions. **B:** Distribution of known GCase variants across the protein. Amino acids positions are color-coded according to the occurrence of variants classified as severe (red), mild/pathogenic (orange), PD risk factor (pink). Positions with variants of unknown

severity are colored in beige. A detailed view of the LIMP-2 interaction site is depicted with individual variant positions labeled.

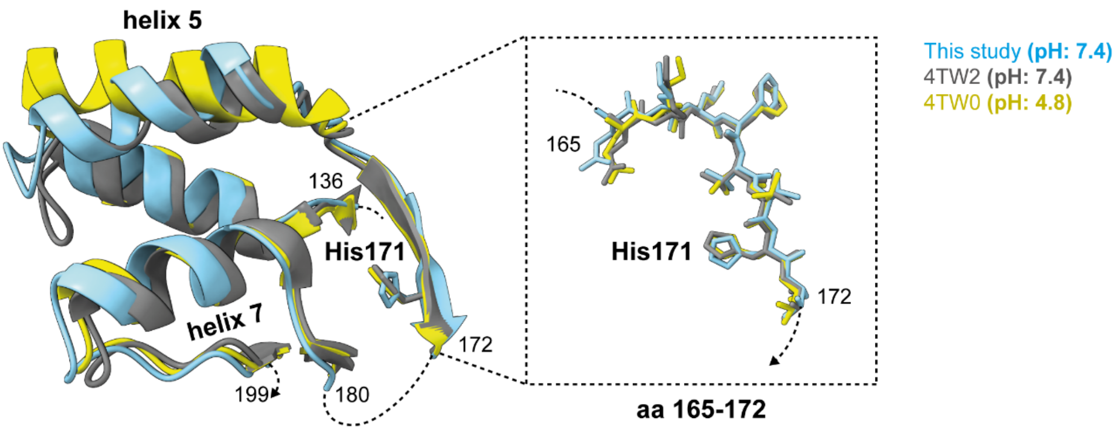

Supplementary Figure 10: Structural comparison of His171 from different structure determination methods and pH values. Structural overlay of His171 and adjacent regions from this study (blue), a crystal structure at pH 7.4 (grey; PDB: 4TW2) and from a crystal structure at pH 4.8 (yellow; PBD: 4TW0) shows very little conformational change between the different conditions.[1]

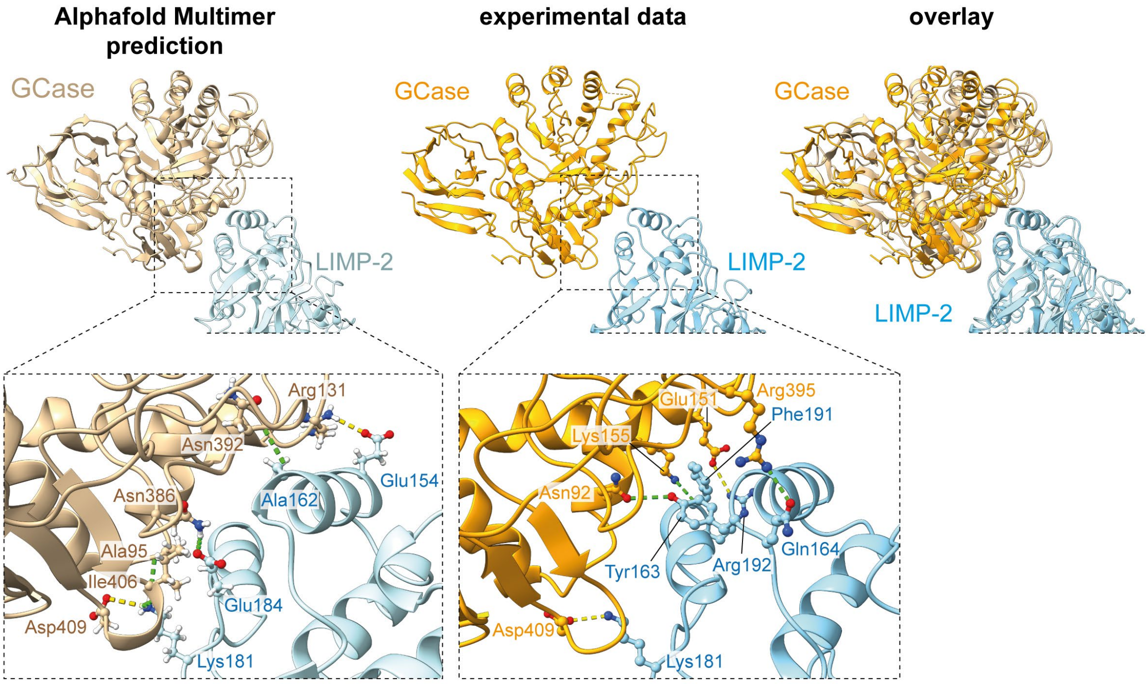

Supplementary Figure 11: Comparison of a LIMP-2/GCase complex structure predicted by *Alphafold Multimer* (left) with the complex experimentally resolved in this study (center). The overlay (right) of both structures shows high similarity between predicted and experimental data. Detailed views (dashed line windows) show the predicted and experimentally determined interfaces with amino acids highlighted that interact via hydrogen bonds (green) or salt bridges (yellow) as determined by PDBePISA. Although the overall structure of the AlphaFold Multimer prediction is very similar to the experimentally determined complex, the interacting residues are inaccurate.

Supplementary Table 1: Hydrogen bonds and salt bridges between GCase and Nb1 (determined by PDBePISA).

Hydrogen bond			Salt bridge		
**GCase**	**Nb1**	**Distance [Å]**	**GCase**	**Nb1**	**Distance [Å]**
Ala168 [O]	Arg115 [NH2]	3.85	His274 [NE2]	Asp62 [OD2]	3.62
Gln169 [O]	Trp116 [NE1]	3.43			
Val172 [O]	Gly109 [N]	2.98			
Lys224 [O]	Tyr111 [N]	3.70			
Gln226 [OE1]	Arg112 [N]	3.88			
His274 [NE2]	Asp62 [OD2]	3.62			
Asn275 [ND2]	Tyr60 [O]	3.88			
Asn275 [OD1]	Lys65 [NZ]	3.73			

Supplementary Table 2: Hydrogen bonds between GCase and Nb6 (determined by PDBePISA).

Hydrogen bond		
**GCase**	**Nb6**	**Distance [Å]**
Phe31 [O]	Thr106 [OG1]	3.48
Ala33 [N]	Tyr107 [O]	3.38
Arg47 [NH2]	Ser56 [OG]	2.25
Glu50 [OE2]	Ser56 [OG]	3.70
Met53 [SD]	Asn32 [ND2]	3.45
Gln57 [NE2]	Arg45 [O]	3.72
Thr61 [OG1]	Glu44 [OE2]	3.83
Thr61 [N]	Glu44 [OE2]	3.90

Supplementary Table 3: Identified GCase-SapC Cross-Links with mass-spectrometry

Interaction Interface	Cross-Linker	Detergent	CSMs	GCase Peptide			SapC Peptide		
				Sequence	Peptide	Position	Sequence	Peptide	Position
Site 1	BS^3	Yes	2	TNGAVNG**K**GLSK	187-198	194	TE**K**EILDAFDKMCSK	24-38	26
	BS^3	No	1	TNGAVNG**K**GSLK	187-198	194	EILDAFD**K**MCSKLPK	27-41	34
	BS^3	No	1	TNGAVNG**K**GSLK	187-198	194	TEKEILDAFD**K**MCSKLPK	24-41	34
	BS^3	Yes	1	TNGAVNG**K**GSLK	187-198	194	EILDAFD**K**MCSKLPK	27-41	34
	BS^3	Yes	1	LFPNTMLFASEACVGS**K**FWEQSVR	336-353	346	EILDAFDKMCS**K**LPK	27-41	38
Site 2	DTSSP	No	1	LL**K**SY	104-108	106	P**K**SLSEECQEVVDTY	40-54	41
	DTSSP	Yes	2	HNFSLPEEDT**K**LKIPL	145-160	155	IDNNKTEKEILDAFD**K**MCSKLPKSL	19-43	34
	DTSSP	Yes	1	PEEDTKL**K**IPL	150-160	157	DAFD**K**MCSKLPKSL	30-43	34
	DTSSP	No	1	ARYFV**K**FLDAY	210-220	215	DAFD**K**MCSKLPKSL	30-43	34
	DTSSP	No	2	DAYAEH**K**L	218-225	224	DKMCS**K**L	33-39	38
	DTSSP	Yes	2	DAYAEH**K**L	218-225	224	DKMCS**K**L	33-39	38

Supplementary Table 4: Hydrogen bonds and salt bridges between GCase and LIMP-2 (determined by PDBePISA).

Hydrogen bond			Salt bridge		
**GCase**	**LIMP-2**	**Distance [Å]**	**GCase**	**LIMP-2**	**Distance [Å]**
Asn92 [OD1]	Tyr163 [OH]	3.83	Glu151 [OE1]	Arg192 [NH1]	3.80
Glu151 [OE2]	Arg192 [NH1]	3.80	Asp409 [OD1]	Lys181 [NZ]	3.47
Lys155 [NZ]	Phe191 [O]	3.79			
Arg395 [NH1]	Gln164 [OE1]	3.40			
Asp409 [OD2]	Lys181 [NZ]	3.47			

Supplementary Table 5: Summary of characterized GCase and LIMP-2 interface variants

Protein	Variant	Type	Affected motif	Reference
GCase	p.E388K	disease-associated (PD)	hydrophilic rim	Parlar et al.,[2]
	p.R395C	disease-associated (GD)	hydrogen bond R395-Q164	Alfonso et al.,[3] Rozenberg et al.,[4] Hruska et al.,[5]
	p.D409H	disease-associated (GD)	Salt bridge D409-K181	Pasmannik-Chor et al.,[6]
LIMP-2	p.Y163C	disease-associated (AMRF)	hydrogen bond Y163 - N92	Uniprot variant viewer
	p.H171A	artificial	pH switch	Zachos et al.,[1]
	p.H171K	artificial	pH switch	Zachos et al.,[1]
	p.K181S	artificial	salt bridge D409-K181	-
	p.F191S	possibly disease associated	hydrophobic core	Uniprot variant viewer
	p.R192S	artificial	salt bridge E151-R192	-

Supplementary Table 6: Primers used to clone GCase and LIMP-2 variants.

Variant	Primer	Sequence 5' – 3'
GCase E388K	Forward	AAAGGAGGACCCAATTGGGTGC
	Reverse	GGGGTTCAGGGCAAGGTTCC
Gcase R395C	Forward	TGTAACTTTGTCGACAGTCCCATCATTG
	Reverse	CACCCAATTGGGTCCTCCTTCG
Gcase D409H	Forward	CACACGTTTTACAAACAGCCC
	Reverse	CTTGGTGATGTCTACAATGATGGG
sLIMP-2 Y163C	Forward	GTCAGCAGAAGCTCTTTGTGACTCAC
	Reverse	AGGCTTTCAACATGGCCTCG
sLIMP-2 H171A	Forward	GCCACAGTTGACGAATTGCTCTGG
	Reverse	AGTCACAAAGAGCTTCTG
sLIMP-2 H171K	Forward	AAGACAGTTGACGAATTGCTCTGG
	Reverse	AGTCACAAAGAGCTTCTG
sLIMP-2 K181S	Forward	GCGATGAAATCTTGTCCCTTATCCATG
	Reverse	TGTAGCCCCAGAGCAATTCG
sLIMP-2 F191S	Forward	CCAGGCCCGATATCTCTCC
	Reverse	AAACATGGATAAGGGACAAGATTTCATC
sLIMP-2 R192S	Forward	CCCCGATATCTCTCCCTATTTTGG
	Reverse	CTGAAAACATGGATAAGGGACAAG

Supplementary Table 7: Summary of Cryo-EM data collection, refinement and validation statistics

	GCase/LIMP-2/Nb1/Nb6 tetramer			
**Data collection**				
Accession (EMDB)	EMD-50936			
Magnification	130,000			
Voltage [kV]	200			
Dose [e/Å^2]	50			
Pixel size [Å]	0.925			
Defocus range [µm]	-0.6 to -2.0			
Recorded movies	10,550			
Final particle images	397,385			
Microscope	Glacios			
Camera	Falcon 4			
Energy filter	Selectris			
**Image processing**				
Initial models [PDB]	GCase: 6TN1; LIMP-2: 4Q4F, Nb1: 9ENA			
Processing Software	cryoSPARC (v.4)			
Symmetry	C1			
**Map**	Non-uniform refinement	Local refinement GCase	Local refinement LIMP-2	Combined map
Resolution (FSC 0.143) [Å]	3.7	3.1	3.0	-
Applied B-factor	-152.7	-143.8	-130.5	-
**Model**				
Accession (PDB)	9J9F			
**Validation**				
FSC map-model (0.143) [Å]	3.6	3.4	3.4	3.2
MolProbity score	1.58			
Clash score	4.92			
**Composition**				
Chains	11			
Atoms	9321			
Protein residues	1124			
Water	27			
Ligands	BMA: 2			
	NAG: 19			
	MES: 4			
	MAN: 2			
**Bonds (R.M.S.D)**				
Length (Å)	0.008			
Angles (°)	1.019			
**ADP (B-factors)**	[min/max/mean]			
Protein	10.01/150.89/70.91			
Ligand	51.02/156.12/100.43			
Water	33.58/76.69/57.24			
**Ramachandran plot (%)**				
Outliers	0.00			
Allowed	4.68			
Favored	95.32			
Rotamer outliers (%)	0.00			

Supplementary References

1 Zachos, C., Blanz, J., Saftig, P. & Schwake, M. A Critical Histidine Residue Within LIMP-2 Mediates pH Sensitive Binding to Its Ligand β-Glucocerebrosidase. *Traffic* **13**, 1113-1123 (2012). https://doi.org/10.1111/j.1600-0854.2012.01372.x

2 Parlar, S. C., Grenn, F. P., Kim, J. J., Baluwendraat, C. & Gan-Or, Z. Classification of GBA1 Variants in Parkinson's Disease: The GBA1-PD Browser. *Movement Disorders* **38**, 489-495 (2023). https://doi.org/https://doi.org/10.1002/mds.29314

3 Alfonso, P. *et al.* Mutation analysis and genotype/phenotype relationships of Gaucher disease patients in Spain. *J Hum Genet* **52**, 391-396 (2007). https://doi.org/10.1007/s10038-007-0135-4

4 Rozenberg, R., Fox, D. C., Sobreira, E. & Pereira, L. V. Detection of 12 new mutations in Gaucher disease Brazilian patients. *Blood Cells Mol Dis* **37**, 204-209 (2006). https://doi.org/10.1016/j.bcmd.2006.09.004

5 Hruska, K. S., LaMarca, M. E., Scott, C. R. & Sidransky, E. Gaucher disease: mutation and polymorphism spectrum in the glucocerebrosidase gene (GBA). *Hum Mutat* **29**, 567-583 (2008). https://doi.org/10.1002/humu.20676

6 Pasmanik-Chor, M. *et al.* The glucocerebrosidase D409H mutation in Gaucher disease. *Biochem Mol Med* **59**, 125-133 (1996). https://doi.org/10.1006/bmme.1996.0077
