# Supplementary material for: Cryo-TEM structure of β-glucocerebrosidase in complex with its transporter LIMP-2
Source: Nat Commun. 2025 Mar 30;16:3074. doi: 10.1038/s41467-025-58340-1 (PMC11955523; doi:10.1038/s41467-025-58340-1)
Supplement: Supplementary file 2 — Reporting Summary [file 41467_2025_58340_MOESM2_ESM.pdf]

nature portfolio

Corresponding author(s): Friederike Zunke and Philipp Arnold

Last updated by author(s): Mar 3, 2025

Reporting Summary

Nature Portfolio wishes to improve the reproducibility of the work that we publish. This form provides structure for consistency and transparency in reporting. For further information on Nature Portfolio policies, see our Editorial Policies and the Editorial Policy Checklist.

Statistics

For all statistical analyses, confirm that the following items are present in the figure legend, table legend, main text, or Methods section.

n/a	Confirmed	
☐	☒	The exact sample size (*n*) for each experimental group/condition, given as a discrete number and unit of measurement
☐	☒	A statement on whether measurements were taken from distinct samples or whether the same sample was measured repeatedly
☐	☒	The statistical test(s) used AND whether they are one- or two-sided<br>*Only common tests should be described solely by name; describe more complex techniques in the Methods section.*
☐	☒	A description of all covariates tested
☐	☒	A description of any assumptions or corrections, such as tests of normality and adjustment for multiple comparisons
☐	☒	A full description of the statistical parameters including central tendency (e.g. means) or other basic estimates (e.g. regression coefficient) AND variation (e.g. standard deviation) or associated estimates of uncertainty (e.g. confidence intervals)
☒	☐	For null hypothesis testing, the test statistic (e.g. *F*, *t*, *r*) with confidence intervals, effect sizes, degrees of freedom and *P* value noted<br>*Give P values as exact values whenever suitable.*
☒	☐	For Bayesian analysis, information on the choice of priors and Markov chain Monte Carlo settings
☒	☐	For hierarchical and complex designs, identification of the appropriate level for tests and full reporting of outcomes
☒	☐	Estimates of effect sizes (e.g. Cohen's *d*, Pearson's *r*), indicating how they were calculated

Our web collection on statistics for biologists contains articles on many of the points above.

Software and code

Policy information about availability of computer code

Data collection	Cryo-TEM movies were collected in a Thermo Fisher Glacios microscope applying the EPU software (v.2.9).
Data analysis	TEM data was analyzed with cryo-SPARC (v.4.2), fitting of existing crystal structure was done with WinCoot (v.0.9.8.7) and Phenix (v.1.19) using ChimeraX (v.1.7.1). Conservation plots were created with BioEdit (v.7.2.5) and sequence alignments with Clustal Omega (v.1.2.4).

For manuscripts utilizing custom algorithms or software that are central to the research but not yet described in published literature, software must be made available to editors and reviewers. We strongly encourage code deposition in a community repository (e.g. GitHub). See the Nature Portfolio guidelines for submitting code & software for further information.

Data

Policy information about availability of data

All manuscripts must include a data availability statement. This statement should provide the following information, where applicable:

- Accession codes, unique identifiers, or web links for publicly available datasets
- A description of any restrictions on data availability
- For clinical datasets or third party data, please ensure that the statement adheres to our policy

Map and model were deposited in the Electron Microscopy Data Bank (EMDB) and the protein databank (PDB). The PDB ID is 9FJF. The respective EMDB ID is EMD-50502. Mass spectrometry data is available under the accession code PXD058056 at the ProteomeXChange Server.

Research involving human participants, their data, or biological material

Policy information about studies with human participants or human data. See also policy information about sex, gender (identity/presentation), and sexual orientation and race, ethnicity and racism.

Reporting on sex and gender	does not apply
Reporting on race, ethnicity, or other socially relevant groupings	does not apply
Population characteristics	does not apply
Recruitment	does not apply
Ethics oversight	does not apply

Note that full information on the approval of the study protocol must also be provided in the manuscript.

Field-specific reporting

Please select the one below that is the best fit for your research. If you are not sure, read the appropriate sections before making your selection.

☒ Life sciences ☐ Behavioural & social sciences ☐ Ecological, evolutionary & environmental sciences

For a reference copy of the document with all sections, see nature.com/documents/nr-reporting-summary-flat.pdf

Life sciences study design

All studies must disclose on these points even when the disclosure is negative.

Sample size	We initially picked ~900k particles and included ~400 particles into the final reconstructuion. Particle number is sufficient to reach near atomic resolution
Data exclusions	Particles showing an incomplet protein complex were excluded from further processing bassed on class sum images.
Replication	The protein complex was enriched multiple times via size exclusion chromatography and the peak shown in the MS was reproduced.
Randomization	We did not randomize samples, as not feasible for experimental setup.
Blinding	We did not perform blindfolded analyses, as not feasible for experimental setup.

Reporting for specific materials, systems and methods

We require information from authors about some types of materials, experimental systems and methods used in many studies. Here, indicate whether each material, system or method listed is relevant to your study. If you are not sure if a list item applies to your research, read the appropriate section before selecting a response.

Materials & experimental systems

n/a	Involved in the study	
☐	☒	Antibodies
☐	☒	Eukaryotic cell lines
☒	☐	Palaeontology and archaeology
☒	☐	Animals and other organisms
☒	☐	Clinical data
☒	☐	Dual use research of concern
☒	☐	Plants

Methods

n/a	Involved in the study	
☒	☐	ChIP-seq
☒	☐	Flow cytometry
☒	☐	MRI-based neuroimaging

Antibodies

Antibodies used	goat anti LIMP-2 (polyclonal, ThermoFisher Scientific Inc., Waltham, MA, United States, #PA5-19111; dilution: 1:1,000), mouse anti GCase (monoclonal, clone E2E, Abnova, Taipeh, Taiwan, #H00002629-M01; dilution: 1:1,000), rabbit anti GCase (polyclonal, Sigma-Aldrich, St. Louis, MO, United States, #G4171; dilution: 1:1,000). Anti MBP Secondary: IRDye 800CW donkey anti goat (LI-COR

	Biosciences, Lincoln, NE, United States, #926-32214; dilution: 1:10,000)
Validation	All antibodies were used and tested against recombinantly expressed proteins. The protein idendity was confirmed via sequencing of the expression plasmid. In cell culture experiments untransfected controls were used.

Eukaryotic cell lines

Policy information about cell lines and Sex and Gender in Research

Cell line source(s)	HEK 293F (ThermoFisher Scientific Inc., Waltham, MA, United States, #R79007), HEK293T cells
Authentication	The cell lines were not authenticated
Mycoplasma contamination	Cells are regularly tested for mycoplasm contamination, none of the tests returned positive
Commonly misidentified lines (See ICLAC register)	none

Plants

Seed stocks	does not apply
Novel plant genotypes	does not apply
Authentication	does not apply
